# ‘That’s how we help each other’: Community savings groups, economic empowerment and HIV risk among female sex workers in Iringa, Tanzania

**DOI:** 10.1371/journal.pone.0199583

**Published:** 2018-07-05

**Authors:** Andrea Mantsios, Catherine Shembilu, Jessie Mbwambo, Samuel Likindikoki, Susan Sherman, Caitlin Kennedy, Deanna Kerrigan

**Affiliations:** 1 Department of Health, Behavior and Society, Johns Hopkins Bloomberg School of Public Health, Baltimore, MD, United States of America; 2 Department of Psychiatry and Mental Health, Muhimbili University of Health and Allied Sciences, Dar es Salaam, Tanzania; 3 Department of International Health, Johns Hopkins Bloomberg School of Public Health, Baltimore, MD, United States of America; 4 Department of Sociology, American University, Washington, D.C., United States of America; Simon Fraser University, CANADA

## Abstract

Female sex workers (FSW) are a socially and economically marginalized population heavily affected by HIV. Community empowerment approaches focus on FSW taking collective action to address structural barriers to their health and have demonstrated effectiveness in reducing HIV risk. This study examines the potential for community savings groups (locally called *michezo*) among FSW in Iringa, Tanzania to reduce HIV risk and promote economic and community empowerment. We conducted 27 in-depth interviews (IDIs) with 15 FSW over time and 4 focus group discussions (FGDs) with 35 FSW participating in *michezo*, and 10 key informant interviews (KIIs) with group collectors. Content analysis was used to identify salient themes around participants’ sex work and financial realities; the role of savings groups in their lives and work; and experiences with social cohesion associated with group participation. *Michezo* were described as providing a safety net for times of financial need, allowing FSW to create greater financial security for themselves and their families. Participation in the groups was also reported to facilitate both individual agency, resulting in members' ability to negotiate condom use and be selective about clients, and a sense of collective identity and solidarity. Participants described group challenges including high mobility and low income of FSW making it difficult for women to fulfill their financial obligations to the group. As a result, participants reported preferences for joining *michezo* whose members were perceived as more stable (e.g. older, married, from more established venues). Group collectors and members were eager to have *michezo* formally registered and become recognized by the broader community. Findings indicate that savings groups promote individual agency to reduce sexual risk behaviors and foster community empowerment among FSW. The groups hold potential as an empowerment strategy to enable sex workers to address structural sources of HIV vulnerability and help them achieve socioeconomic inclusion.

## Introduction

Female sex workers (FSW) are a socially and economically marginalized population heavily affected by HIV. Globally, it is estimated that they have 13.5 times greater odds of having HIV than other adult women [1]. HIV prevalence among FSW in sub-Saharan African countries is significantly higher than in other geographic regions, with an estimated pooled prevalence of 36.9% [2]. In the Iringa region of Tanzania, a recent integrated biobehavioral surveillance survey found that 32.9% of FSW were living with HIV [3]. Across geographic settings, FSW are a population often living in poverty and balancing competing financial priorities such as food, housing, children’s expenses, and medical costs [4–7]. Economic realities for FSW can include low education and lack of skills for formal employment, scarcity of jobs, and low pay [8, 9]. Social and structural factors such as financial insecurity, stigma and discrimination, violence, and legal and policy environments surrounding sex work have all been linked to heightened HIV risk of FSW[10].

Given the established literature indicating the importance of financial insecurity as a driver of HIV risk behaviors such as unprotected sex among FSW [11–18], economic interventions that aim to promote financial security have gained traction [19]. Strategies for increasing financial security of FSW can include microfinance, vocational training and income generating activities, cooperative banking, and savings and money management. Which of these strategies an intervention chooses to focus on reflects a fundamentally different understanding of and approach to economic empowerment of FSW. Namely, focusing on securing alternative income can be conflated with efforts to rescue or rehabilitate women from sex work while focusing on the role of savings and money management can promote financial security without intervening on a woman’s decision to engage in sex work. Having women leave sex work is not necessary to improving the economic conditions of their lives. Focusing on increasing financial security with the income they have from sex work is rooted in a rights-based approach in which sex work is recognized as work.

While the literature on economic empowerment interventions with FSW is limited [20, 21], promising models are provided by programs that have implemented economic components within a broader community empowerment approach. A community empowerment framework is one in which FSW take collective ownership of strategies to address structural barriers to their health and human rights [22]. Durbar [23–26] and Pragati [27, 28] are two programs in India that model how economic empowerment can occur within the context of promoting social cohesion and collective identity among FSW communities. By situating economic interventions within a community empowerment approach, these programs focus on FSW addressing economic issues alongside, and as part of, empowerment strategies to address other structural vulnerabilities including stigma, discrimination, violence, and social inequality [24, 26]. The focus of such programs is thus on FSW taking collective action to confront their economic marginalization as a community, rather than on increasing individual income alone. Only a handful of such programs can be found in the literature. Other examples and further evaluation of community empowerment-based economic approaches are needed, particularly in the context of sub-Saharan Africa where HIV burden among FSW is greatest.

Our understanding of community empowerment is informed by sociologist Anthony Giddens’ structuration theory [29], which proposes a duality in which an individual’s agency is influenced by social structure while societal structures are, in turn, simultaneously maintained and adapted through the exercise of individual agency. Through this lens, we recognize the role of contextual factors of sex workers’ lives and the critical role social structure plays in determining their health choices, but at the same time we recognize the collective ability of sex workers to come together to reshape structural constraints [30]. It is through a community empowerment process that FSW can gain individual and collective agency to effectively address power imbalances and the structural sources of their HIV vulnerability.

We have also situated our research within the theoretical orientation of social and economic exclusion [31] to conceptualize the processes by which FSW, as a historically marginalized population, experience structural constraints to their health and rights. Social and economic exclusion entail a lack of opportunities, blocked access to resources and services, and marginalization from decision-making in society and can be understood as a group-level form of discrimination [32, 33]. FSW face multiple forms of stigma and discrimination. Their marginalized status coupled with low education and literacy levels present significant barriers to their ability to access traditional financial institutions, economic activities, and the labor market [34]. For FSW, the interaction of social and economic exclusion limits their access to resources necessary to protect their health. Specifically, in the case of HIV, economic exclusion plays into financial insecurity and economic vulnerability to infection. Economic vulnerability includes not only poor economic status but also financial debts, control over resources, lack of other livelihood options, as well as food insecurity [14, 20, 35]. Community empowerment, and economic empowerment within that process, introduce promising strategies for overcoming the complex interaction between social and economic exclusion faced by FSW.

Qualitative research previously conducted in Iringa revealed organically formed community savings groups (locally called *michezo*; singular: *mchezo*) among FSW [36]. The current study explores the potential role of these groups in reducing economic vulnerability to HIV among FSW in Iringa and in serving as a mechanism through which FSW can take collective action towards greater social and economic inclusion. This qualitative research served as formative work for an economic empowerment component within a Phase II community-based combination HIV prevention trial called Project Shikamana conducted among Tanzanian women at heightened risk in Iringa.

## Methods

### Study setting

The Iringa region is bifurcated by a major transport corridor, the Tanania-Zambia highway. Truckers traveling the highway often spend multiple days traversing the region, thus there are guesthouses, bars, restaurants, gas stations, weigh stations and truck stops in the larger villages and small towns along the route. Many of these stops also function as sex work venues, and truckers comprise a large portion of FSW clientele. There are also seasonal fluctuations of sex workers in and out of the region that mirror the seasonal fluctuations of migrant laborers working on the plantations and farms in the region. Most sex work in Iringa is venue-based, with establishments located along the highway and in the towns along the route [37]. There are generally two distinct types of bars where FSW work–“vilabu” which tend to be smaller and serve locally brewed alcohol and “modern bars” which tend to be larger and sell bottled beer and liquors.

### Recruitment

Between April 2015 and February 2016, we conducted 27 in-depth interviews (IDIs) with 15 FSW over time and 4 focus group discussions (FGDs) with 35 FSW whoc participate in *michezo*, as well as 10 key informant interviews (KIIs) with group collectors, those tasked with collecting and holding the group money, in the Iringa region. Inclusion criteria included women 18 years and over who reported exchanging sex for money in the last month. We purposively sampled for women participating in savings groups and sought a diverse sample with regards to age to allow for exploration of themes relating to a life course perspective (e.g. decreasing sex work income with increasing age) and HIV status to explore how financial insecurity and savings group participation may play out differently for HIV-infected women (e.g. having resources to pay transport costs for HIV-related clinic visits). Women who reported participating in *michezo* in a previous study conducted by our team were first recruited into the study to participate in IDIs. Then, using snowball sampling, study participants were asked to recommend other FSW who participate in *michezo* for IDIs and FGDs; however due to confidentiality, peer referral according to HIV status was not sought. This resulted in a sample of majority (11/15) HIV-infected women for the IDIs. Ten women who serve as group collectors were recruited to participate in KIIs by asking study participants to refer their group collectors.

Interviews took place at or near the study participant’s work or home, based on her preference, and lasted approximately one hour. A semi-structured interview guide was used to gather information on key domains within financial security, sex work, and participation in community savings groups including questions around how group participation affected women’s work. Of the 15 FSW who initially participated in IDIs, 12 completed a follow-up interview 8–12 weeks later. The second interview provided an opportunity to revisit some of the topics discussed with the intention that existing rapport with the interviewer would facilitate further depth of information, particularly around sensitive topics such as personal finances.

Four FGDs were conducted with 35 FSW who participated in *michezo*. FGDs were intended to facilitate an understanding of norms, expectations and local conceptualization of the concepts of interest in the study [38, 39]. The goal of exploring similar topics covered in the interviews with individuals in the group setting was to discover how FSW think and talk about these issues and generate insight into their shared understanding of their lives, culture, and experiences [39]. FGDs took place at centrally located meeting spaces that had a confidential area for group dialogue. Each group had between 7–11 women and lasted approximately 90 minutes. A discussion guide was developed outlining key domains, but the facilitator was encouraged to probe and explore related topics and experiences.

Group collectors, women in leadership roles in *michezo*, were interviewed for two reasons. First, collectors clarified operational aspects of the groups given that they were more intimately involved in setting and managing group rules, dynamics, and challenges. Second, they provided the perspective of women in the highly trusted position of collecting and holding the group money. An interview guide was developed to elicit responses to questions about group functioning and visions for the future of the group while allowing for the participants to also provide their perspectives on the meaning and importance of *michezo*.

### Sample characteristics

As shown in Table 1, IDI and FGD participants ranged from 20 to 45 years old, with a mean age of 29. Among the sample, 80% (40/50) of the women were single, 9 women were married or reported a permanent partner, and one woman was a widow. Nearly all participants (90%) had children and over half (56%) had 2 or more children. Education levels were low with 38% (19/50) having some secondary school, 60% (30/50) having primary-level education and one individual who had no schooling. Of the 15 FSW who participated in IDIs, 11 were HIV-infected. HIV status of FGD participants was not collected to maintain confidentiality. The 10 group collectors who participated in KIIs ranged in age from 22–32 years old, with a mean age of 28. Eight of the collectors had primary level education and 2 had some secondary schooling.

**Table 1:** Sociodemographic characteristics of IDI and FGD participants.

**IDI and FGD Participant Characteristics (N = 50)**	**Median [range]** **or N (%)**
**Age**	29 [20–45]
**Martial status**	
*Single*	40 (80%)
*Married/permanent partner*	9 (18%)
*Widow*	1 (2%)
**Children**	
*Any children*	45 (90%)
*≥ 2 children*	28 (56%)
**Education**	
*Some secondary school*	19 (38%)
*Primary-level education*	30 (60%)
*No schooling*	1 (2%)
**HIV-status**^**a**^ (N = 15)	
*HIV infected*	11 (73%)
*HIV uninfected*	4 (27%)
**Collectors Participant Characteristics (N = 10)**	**Median [range]** **or N (%)**
**Age**	28 [22–32]
**Education**	
*Primary school*	8 (80%)
*Some secondary school*	2 (20%)

^a^Not collected for FGD participants

### Data collection

All data collection was conducted in Swahili by a female, Tanzanian study staff member trained in qualitative research methods who had extensive experience working with FSW in Iringa. Debriefing sessions between the first author and the interviewer/facilitator were conducted following each of the IDIs, FGDs, and KIIs. These sessions facilitated an iterative process of data collection and analysis and provided guidance for topics to explore further in the follow-up interview with each participant and in subsequent FGDs. All interviews and focus groups were audio recorded, transcribed, and translated into English. Oral informed consent, rather than written consent, was obtained from all participants prior to participation in the study based on the highly stigmatized nature of the study population. Oral consent was documented by the interviewer’s signature upon witnessing the participant agree to participate.

To maintain participant confidentiality, only a coded participant identification number was used on all study data including recordings and transcripts. Access to all study data files was password protected and all data collection instruments and study documents were stored in locked file cabinets.

### Data analysis

Qualitative analysis was conducted using an iterative thematic analysis approach both drawing on *a priori* codes and allowing for emergent codes and themes [40, 41]. Memos were developed from multiple readings of each transcript to assist in development of salient themes. The memos were used throughout data collection to document thoughts about the significance and relationships of codes to one another and note questions that arose from the coding process [42]. Journaling throughout the study allowed the first author to reflectively engage with the research and be cognizant of the biases she was bringing to the analysis. Processing these biases with the interviewer/facilitator throughout the study kept reflexivity [43] central to the work. A codebook was developed based on the themes emerging from the data. Coding output was synthesized across key domains, categories were identified, and codes were arranged hierarchically with sub-codes listed under major categories. Major categories under which codes were arranged included trust, support, stigma and solidarity.

Different approaches were used to analyze IDI, FGD, and KII transcripts. IDIs were analyzed in the tradition of a narrative approach, placing value on the women’s storytelling of their lives in sex work and their financial realities, used as an opportunity to reveal cultural and social patterns through the lens of individual experiences [41]. FGDs were analyzed as a collective dialogue in which the group itself was the unit of analysis and the group dynamics and interpretations and meaning of what participants shared was understood in the context of the larger group. Analysis focused on interpreting a collective view of participants’ understanding of the world rather than treating them as a compilation of the views of different participants in the group [39]. Analysis of KIIs included coding for operational codes to identify key components and functions of the groups. All interview and focus group transcripts were coded using ATLAS.ti qualitative data management and analysis software [44].

This study received human subjects research approval from the Institutional Review Boards of the Johns Hopkins University Bloomberg School of Public Health, Muhimbili University of Health and Allied Sciences, and the National Institute for Medical Research of Tanzania.

## Results

Building on prior formative work in Iringa which revealed the use of *michezo* among FSW in the region [36], the current study clarified savings group structure and operations. *Michezo* were described as involving a rotating payout in which each member receives the total amount pooled from all members’ regular contributions. At the start of the cycle, all members choose a number that dictates when in the cycle they will receive the rotating payout. Each member contributes the group’s pre-specified amount of money on a regular basis. The payout of the lump sum is given to the member who has the next sequential number in the cycle. The length of the cycle is determined by how many members are in the group. The amount of the regular contribution can be determined by the collector or decided upon by the group.

Key findings described in detail below include the organically formed and community established nature of the groups, the ways in which women felt that *michezo* provided them with financial and social support, the sense of solidarity and collectivism women felt from participating in *michezo*, and how group participation affected women’s HIV risk. Some of the tensions and challenges experienced within the groups included a perceived sense of exclusion of certain women from participating and the desire women expressed to have the groups formally registered and become recognized by the broader community.

### Community established and led

Participants described *michezo* as forming organically among community members with varying composition–women only, exclusively FSW often organized within sex work venues, or mixed community members including both men and women. Participants explained, however, that when FSW joined *michezo* of mixed community members, it often became clear they were not welcome, leading many participants to join or form FSW-only groups. Participants described FSW *michezo* as operating covertly, due to fear of stigma from the non-FSW community. One participant said, “We will keep it to ourselves that it’s *mchezo* for *dada poa* [FSW; literally “cool sisters”] only; it will only be known in our community…we will give it a different name …but deep inside we know that it’s a *dada poa* only group” (IDI participant, age 39). Another participant described:

*It’s true we can never invite someone from the regular community because they will expose us*. *We are very good at keeping secrets*. *If we get someone else from the regular community*, *automatically she will know we are sex workers*. *They will sit down and talk about us; she will expose how we run our business*. *That can bring problems in the neighborhood*. *That’s why we chose [to include only] ourselves*, *because we know we can keep our secrets*.—IDI participant, age 30

Participants spoke specifically about the unique challenges they faced as sex workers and the benefit of coming together as a community to support their future livelihoods. Recognizing the occupational realities of HIV risk and aging out of sex work, one woman who started a group with her colleagues explained:

*I called [my fellow FSW] and we sat at the table*. *I told them these jobs have an end*. *Where we are going to get men*, *there is AIDS*, *it may reach a point when you lose all the power to work*. *It may reach a point when you will be worn out*, *even the men won’t desire to sleep with you*. *In that sense*, *if we participate in a* mchezo *you can get money*. *You may get money and do something meaningful; you may even buy a plot and build a small house of one room*. *Why don’t we participate in a* mchezo? *They all saw this was a good idea*.*—*IDI participant, age 30

Women described involvement in group decision-making and adapting the groups to the changing needs of members. One participant recounted the story of when her group decided to increase regular contributions to meet their increasing needs, saying, “I was part of that decision. Our collector involved all of us in it. We discussed it and decided to increase the amount from 1,000 to 2,000 because life has changed and now people have a lot more needs” (IDI participant, age 34). A group collector discussed the need for the groups to maintain a flexible structure in which members decided if and when the group needed to change saying, “*if a problem happens*, *we members should focus and adapt; days are moving forward and life changes…when we change the form of the* mchezo, *we have to assess what we will do as a group* …*if we get problems*, *we must cooperate…we build team cooperation”* (Group collector, age 25).

### Financial and social support

Participants saw *michezo* as necessary to being able to financially support themselves and their families. The group payouts were considered an essential supplement to sex work income that allowed participants to be able to afford basic needs such as daily food staples, rent, school fees, clothing for their children, and housewares. Others saw money from *michezo* as a way to save for large purchases or investments for the future. Many participants spoke about saving money to purchase a plot of land or build a new home, longer-term plans they spoke of in the context of financial aspirations they felt could only be attained by participating in *michezo*. Speaking about the role of *michezo* in the lives of sex workers and, more broadly, the potential for improving one’s living conditions, one participant said:

*The advantage of a* mchezo *is that you can improve your financial stability very fast compared to when you save money yourself*. *I cannot live without a* mchezo*; any sex worker cannot live without a* mchezo*…A* mchezo *can help you afford to do a lot of things*, *big things*. *When you look at sex workers who are not in a* mchezo, *it’s very difficult for them to improve their living standard; they will never improve*.***–***IDI participant, age 30

Participating in *michezo* was also described as providing an insurance mechanism in times of need, which left women feeling less vulnerable to financial crises. An important feature of the groups was the ability of members to switch places in the rotation with another member when experiencing particular financial strain. In addition to the value of receiving monetary support, many participants described this aspect of the groups as tied to a sense of emotional support they felt from other members assisting them with their problems. As one participant described:

*That’s how we help each other*, *not because it’s your turn then and you want to just take the money without caring about your friends and their problems*. *We listen to each other*, *and we listen to our friends’ problems*, *how big their problems are and how we can help them*.–IDI participant, age 30

### Solidarity and collectivism

Many participants described the groups as a network of people who care for one another in times of need, including collecting additional money to help members cover the cost of unexpected hospital bills, medication, and funerals, or even providing support for cooking and other household chores when another member falls ill. One participant described *michezo* as fundamental to how problems are handled in the community, saying, “it means when you get a problem, the group is obligated to help you because you are one of them…we live by cooperating with each other” (IDI participant, age 35). Participants made it clear that camaraderie within the sex worker community over their shared experiences fueled a sense of solidarity within the savings groups. One collector spoke about the need for *michezo* in order for FSW to be able to help each other through the challenges they faced in their work:

*In our group*, *we sat and thought*, *because we all do our activities differently*. *And it happens someone may go to her activities and face problems*. *You find she comes back with no money at all*. *There is this and that problem*, *so we help her…we help each other*. *As a group member who has a problem*, *we need to do something to help her*. *We know the whereabouts of one another*. *So if I go any place and I am harmed*, *then I just get in touch with my fellows*. *One will come or maybe send a motorcycle*. *It’s like a certain type of union*. *We decided to form our own* mchezo *because of this business that we are doing*.*—*Group collector, age 30

The importance of having a common understanding of shared experiences among FSW within *michezo* was evident in the ability to relate to and support each other through challenges with clients who refused to pay for services or experiencing violence from clients. One woman said:

*Maybe you went with a client and he hit you pretty hard*. *Then you’re just sick at home…you have nowhere to go*, *no one to talk to*. *You don’t know where to go*, *you don’t have money…*.*because we know each other*, *we can take her to the hospital to get treatment*.*—*IDI participant, age 39

Women whose savings groups had regular meetings described the meetings as opportunities for information sharing, exchanging advice, and addressing group dynamics and community issues. One collector shared that she had begun providing group members with advice on money management. Another collector reported that sexual health advice was shared during meetings, including encouraging condom use with clients and HIV testing. One group member spoke about the challenge of sex work clients seeking lower prices at different venues in the area and reported that it was during savings group meetings that members would discuss and set the prices they thought should be used by FSW across local venues.

### Group participation and HIV risk

Participants reported feeling that they had little or no power to negotiate the terms of sex with clients when they knew they did not have money to meet their basic needs. However, they unanimously reported that participating in *michezo* created a safety net they utilized when they had immediate financial need, thus allowing them to refuse high-risk sex with clients. Many women articulated that knowing they had money from *michezo* allowed them to feel in control of being able to have safer sex with clients. They described a sense of agency to participate in decision-making in their interactions with clients which allowed them to choose when, with whom, and for how much they would provide their services. One focus group participant captured the group dialogue around this theme when she described how her *mchezo* impacted her ability to face condom negotiation challenges with clients:

*He will tell you*, *‘I cannot have sex with you using condoms*, *I want it without a condom*.*’ A* mchezo *helps you avoid these kinds of challenges in our work; you will be able to tell a client*, *‘If you cannot use a condom*, *I’m sorry*, *I will have to leave*.*’ But if you don’t have money and you don’t know where else you will get money for food*, *you will agree to have sex*, *even without a condom*.–FGD participant

Another participant articulated the sentiment of her focus group around the difference *mchezo* participation can make in decision-making around unprotected sex with clients by saying:

*The mchezo has helped me a lot*. *For example*, *you might get a client*, *and he will refuse using a condom*. *But I can decide to refuse because I know even though he doesn’t pay me*, *I have money at home from the* mchezo. *It’s different from when you’re not in a* mchezo, *you might just go without a condom because you want money*. *But now I make my own decisions*.–FGD participant

A number of women reported that when they had money from *michezo*, they engaged in price negotiations with clients. Crystallizing this dynamic described by many, one focus group participant asserted to the group:

*I will tell the customer that 30*,*000 Tsh is the price for the service and I mean it*, *because I know I have money at home*. *For instance*, *if I am used to having sex without a condom and on that day I tell him we should use a condom*, *if he disagrees*, *then that’s going to be end of the story* [laughs].–FGD participant

Another participant reported providing clients with a set of terms of her services when she knew she was not dependent on them for money:

*When my customer arrives*, *I start negotiations at a higher price*. *Let’s say the normal price is 20*,*000 to 50*,*000 Tsh*, *then I start with 100*,*000 Tsh*, *just because I have money [laughs]*. *He will ask himself*, *‘Why is this person different today*?*’ And that’s where I give him my rules*. *So when he agrees with my terms*, *then we can get to business*.—FGD participant

### Tensions and group exclusivity

While group dynamics were often described as fostering solidarity and support, participants also described tensions and exclusivity within the FSW community that presented barriers to cohesive FSW *michezo*. A few participants said that strained interpersonal dynamics between FSW peers (e.g. competition for clients, quarrels between coworkers at a venue) could be problematic. However, participants overwhelmingly focused their group grievances on the characteristics of their FSW peers that made them undesirable as group members and resulted in exclusion of certain women from the groups. Multiple study participants expressed frustration with fellow FSW group members due to their mobility and the threat of them leaving mid-way through the payout cycle, leaving other members without their full payout. One participant described:

*Others might run with the money*, *as you know sex workers are mobile; after someone takes the money*, *she runs off*. *Most of them from the big bars do join with those of us from the neighborhood*, *but even us*, *we tend to have doubts…If we give it to her at the beginning [of the rotation]*, *she might run away*. *Most of them [*michezo *groups] keep her as the last one [in the rotation]*. *They [women from the bar] do run away*.*—*IDI participant, age 28

The sentiment captured in this quote that women who work in certain bars were more mobile, and thus less trustworthy, was expressed by numerous participants. Many participants noted that bar maids in “modern bars” (those that sell bottled beer and liquors, as opposed to locally-brewed alcohol) tended to come in and out of the area with the seasonal fluctuations of migrant workers and thus lacked the permanency and reputation of trustworthiness of women working in the local-brew bars. Unfamiliar or unknown FSW who did not have other women to vouch for them were thus seen as undesirable potential group members. One participant described the *michezo* vetting process for sex workers who were not established in the area with a home or sense of permanence. She reported:

*We investigate our members*, *but also our* mchezo*…we all know each other and we trust and understand each other*. *Therefore*, *we must investigate each member who will ask to join*, *and the collector will even ask*, *“My dear friends*, *this person wants to join us; do you trust her*?*” Maybe I will say*, *“I don’t know her*.*” Maybe someone else will say*, *“I know that her house is located at a certain place*,*” so the collector will know she is not mobile*, *she has a permanent home*. *But if she is just a barmaid…unless I guarantee that I will pay for her if she is not around…we don’t allow her*.—IDI participant, age 24

Because of these common perceptions that FSW were more likely to move away during a payout cycle and were less reliable in paying their contributions on time, some participants expressed preference for participating in groups with women they perceived as more “stable,” such as older and married women.

Another challenge for FSW *michezo* was that low and unsteady income could make it difficult for FSW to pay the regular group contribution when it is due. Participants explained that sometimes FSW in the group struggled to meet the regular contribution and this complicated the cycle for everyone. Some collectors said they felt it was best if members had a business or some source of steady income as assurance they would be able to pay into the group. Other collectors said that anyone was welcome as long as they could meet the regular payments. They argued that, with some groups taking a regular contribution of as little as 1,000 Tsh (50 cents), there was “no one” who would not be able to join a group. A number of participants also complained that FSW-only savings groups were too lenient in enforcing on-time contributions. They expressed the view that women tend to “have mercy” on their peers when they were unable to make payments, whereas in savings groups of mixed community members, men were much less tolerant. This was cited as a reason to participate in mixed-member groups rather than a FSW-only *mchezo*.

### Moving towards social and economic inclusion

As participants described exclusion from formal banking due to insufficient income, they explained that *michezo* provided them a mechanism within the community that allowed them to safely save money. Group members and collectors alike expressed that their vision for the future was to move their groups into a more formal capacity. They specifically spoke about their desire to gain recognition, register their savings groups with the government, and achieve social inclusion in the broader non-FSW community. One participant said, “My vision, what I see, is being recognized by the community and the media…We are not known anywhere; everything we are doing has to be done secretly” (IDI participant, age 30).

Many women wanted to register their groups with the government because they believed it would help them enforce payments by members who evade them. One collector said that when someone does not pay, “there is nothing to do because these groups are still small, we can’t take any legal measure because these groups are not registered, which makes it difficult to take someone before the law” (Group collector, age 27). While many women thought that registering their groups with the government would improve group functioning by introducing more formal accountability, others were optimistic that this would help them be recognized and respected by society. One focus group participant spoke of wanting to “do something in society so I can be seen as if I am somebody.” Another participant said:

*Sex work is work like any other work*. *It’s just that we are not recognized*. *Maybe on social media like radio*, *for example*, *here in* Ilula, *we’re not recognized at all*. *I don’t even think people know that we are running a* mchezo *and we are really helping each other*.*–*IDI participant, age 30

When describing their visions for *michezo* to gain more formal recognition and become registered entities, fear of stigma-related challenges that they might face during this process did not arise in interviews or FGDs. Many women spoke with optimism about the future of their groups. Participants reflected on how much their groups had already grown in size and contribution amount. They had clear ambitions for continuing to strengthen and grow the groups and said that registering with the government and becoming formally recognized was a natural next step.

## Discussion

The community savings groups explored in this study promote individual agency to reduce sexual risk behaviors and foster community empowerment among FSW in Iringa. Our findings indicate that *michezo* serve as an economic and psychosocial resource for FSW. More broadly, the groups enable FSW to collectively address structural factors contributing to their HIV risk and vulnerability. These findings are supported by previous quantitative research conducted by our study team which identified a positive association between *michezo* participation and consistent condom use with new clients among FSW in Iringa [45]. The qualitative findings presented here provide an understanding of the pathways through which *michezo* participation contributes to reduced HIV risk behaviors among FSW in Iringa. Participating in *michezo* was found to promote empowered decision-making, enabling women to more effectively navigate condom negotiation and safer sex with clients. These findings echo research from other settings which found that having access to secure savings better positions sex workers to refuse unsafe sex and negotiate condom use, and that sex worker collectives can empower women to more actively negotiate the terms of their work [24, 46, 47].

Beyond enhancing individual agency, *michezo* foster social cohesion and collective agency, which serve as mechanisms through which community empowerment is achieved. This collective agency allows FSW to counter their social and economic exclusion vis-a-vis group status. Returning to the duality of structure Giddens proposes, namely that an individual’s agency is influenced by social structure and social structures are maintained through the exercise of individual agency, FSW are reshaping social structures by exercising agency in creating informal financial institutions. In turn, by reshaping structure, their opportunities and agency to think and act autonomously are transformed. Developing both individual and collective agency allows FSW to reshape and redefine the very structures that constrain them, challenging social order and creating new norms and relationships [29]. The need for social recognition of sex work as work, expressed by some participants, is an important indication that the process is underway for FSW to utilize their collective agency to mobilize and advocate for a voice and presence in the broader community. This highlights the importance of programs adopting a rights-based approach which promotes both individual and community level economic empowerment. Programs must aim to help sex workers achieve the economic power to make informed choices about their lives and to protect their sexual health [21], while also focusing on community empowerment for bringing about social and structural change for the broader FSW community.

The negative aspects of the groups highlighted by study participants raise numerous important questions that warrant further study. Findings indicate that the overall low income and high mobility of FSW in Iringa pose challenges to FSW-only savings groups and fuel exclusion of certain women from the groups. Preferences to participate in *michezo* based on the age and marital status of the group’s members indicate exclusivity of the groups to a subpopulation of FSW. In the aforementioned quantitative study conducted by our study team, we found among a cohort of 496 FSW in Iringa that women who participated in *michezo* were more likely to be older and married [45]. Stigma towards FSW who work at modern bars and are considered transient and therefore untrustworthy as group members suggests discriminatory practices in group membership. Further research is needed to explore how the tensions discussed here create division among FSW and perhaps negatively affect the community empowerment process, and in what ways these groups may be excluding the most marginalized and vulnerable FSW within the community. Future programming to support *michezo* as an economic strengthening approach to reducing HIV risk among FSW in Iringa should focus on strategies for engaging younger women and overcoming barriers faced due to mobility, such as continued engagement in the groups during times of travel outside of the region such as through mobile money payments.

Limitations of this study include the potential bias introduced by using snowball sampling for participant recruitment. This resulted in a majority of HIV-infected women participating in the interviews rather than a more balanced sample however the interview guide was designed to elicit perspectives on effects of group participation on work which provided rich data on reduced risk behaviors across HIV-infected and HIV-uninfected women. Though HIV status was not collected from FGD participants in order to protect confidentiality, it can be assumed that the 35 women who participated included a mix of HIV-infected and HIV-uninfected women, given the 41% HIV prevalence documented in the parent study cohort [48]. Three of the women who participated in initial IDIs were unavailable for a second interview; thus, we were not able to pursue follow-up questions and additional exploration of topics with those participants. By design, this study explored community savings groups with FSW who were participating in *michezo* at the time. It therefore lacks insight from FSW who do not participate in the groups to help understand differences between women who do versus do not participate in the groups, those who stay engaged versus those who leave the groups, and those who may be excluded from the groups. The authors intend to explore this perspective in future research.

## Conclusions

Community savings groups are an accepted element of Tanzanian society and are commonplace in other sub-Saharan African countries and around the world. The groups appear to naturally facilitate HIV risk reduction and offer an existing, sustainable platform for economic empowerment and engagement in HIV prevention for FSW in high prevalence settings. This has implications for the conceptualization of public health programming for FSW and other populations of women at high risk for HIV. In forming community savings groups, FSW in Iringa are working to build financial security and create economic stability for themselves and, in their effort to do so, have exercised agency to reshape the structures that constrain them. Their increased individual and collective agency around HIV decision-making is a result and additional benefit of that process. The savings groups explored here underscore the need for the process of embracing protective behaviors to be stimulated and supported by communities and driven by their intentions to have their identified needs met. HIV prevention efforts should support the formation and growth of *michezo* as a means through which FSW can have their fundamental needs met and through which adoption of protective behaviors can naturally follow. *Michezo* and other types of savings groups should be considered part of a rights-based comprehensive approach to addressing HIV among FSW.
